# Are We Real When We Fake? Attunement to Object Weight in Natural and Pantomimed Grasping Movements

**DOI:** 10.3389/fnhum.2016.00471

**Published:** 2016-09-22

**Authors:** Caterina Ansuini, Andrea Cavallo, Claudio Campus, Davide Quarona, Atesh Koul, Cristina Becchio

**Affiliations:** ^1^C’MON Unit, Fondazione Istituto Italiano di TecnologiaGenova, Italy; ^2^Department of Psychology, University of TurinTorino, Italy; ^3^U-VIP Unit, Fondazione Istituto Italiano di TecnologiaGenova, Italy

**Keywords:** reach-to-grasp, pantomime, object weight, kinematics, linear discriminant analysis

## Abstract

Behavioral and neuropsychological studies suggest that real actions and pantomimed actions tap, at least in part, different neural systems. Inspired by studies showing weight-attunement in real grasps, here we asked whether (and to what extent) kinematics of pantomimed reach-to-grasp movement can reveal the weight of the pretended target. To address this question, we instructed participants (*n* = 15) either to grasp or pretend to grasp toward two differently weighted objects, i.e., a light object and heavy object. Using linear discriminant analysis, we then proceeded to classify the weight of the target – either real or pretended – on the basis of the recorded movement patterns. Classification analysis revealed that pantomimed reach-to-grasp movements retained information about object weight, although to a lesser extent than real grasp movements. These results are discussed in relation to the mechanisms underlying the control of real and pantomimed grasping movements.

## Introduction

The ability to reach out and grasp objects with considerable skill is one of the defining features of primates. In both humans and non-human primates, prehension is typically directed at a visible object and results in contact with the object, manipulation, and haptic feedback. Humans (and perhaps some other species, Douglas and Moscovice, 2015), however, are also capable of grasping an imaginary object. The interest in this ability, both for clinical and theoretical investigation, is due to its double nature (Goldenberg, 2013). Pantomimed actions are derived from instrumental actions of actual use. They are, however, communicative gestures in that they involve the repetitions of instrumental movements, but without acting on an object, as a way of communicating something about the action or the object.

This double nature of pantomime – both instrumental and communicative at the same time – is reflected in the differential use that real and pantomimed grasps make of object knowledge. In real grasps, knowledge about objects and their manipulation is used to conform the hand gradually to the properties of the object to be grasped. For example, when grasping a glass, scaling of grip width to the width of the glass is achieved by first opening the hand in proportion to, but wider than the visually perceived width of the glass, and then closing it around the glass, ensuring a safety margin for grasping the object securely (Jeannerod, 1988; but see also Smeets and Brenner, 1999 for an alternative account of this effect).

In contrast, in pantomimed grasps, knowledge about objects is converted into actions that *demonstrate* the perceptual distinctive features of the pretended objects (Goldenberg et al., 2007). This conversion necessitates the selection of some features of the actual grasp, while permitting one to neglect others, i.e., those features that adapt the hand to the material object. Thus, when pantomiming, for instance, participants do not show grip ‘overshoot,’ but open the hand to the approximate width of the pretended object ‘to depict’ its width (Goodale et al., 1994). One particular difficulty of pantomimed grasp tasks relates, therefore, to the transformation of object features into a non-routine movement sequence that *demonstrates the perceptual features of the pretended object* (Goldenberg et al., 2003).

Although previous research indicates that pantomimed grasp incorporates spatial features of a pretended target, such as its actual (Goodale et al., 1994; Cavina-Pratesi et al., 2011) or visually perceived size (Westwood et al., 2000), it is questionable whether pantomimed grasp can also demonstrate non-spatial features of the target. Specification of object size requires selecting a simple spatial characteristic of the object (e.g., the width of the object) and converting it into a spatial relationship between a limited set of discrete body parts (e.g., the distance between thumb and index). Arguably, depicting a non-spatial characteristic of the object, such as its weight or fragility, might be more complicated as no simple perceptual matching is possible for transforming the representation of the weight or the fragility of an object into a distinctive grasping pattern.

Here we set out to examine the representational reach of pantomime by asking whether pantomimed grasping can transmit information about the weight of a pretended object.

### Influence of Object Weight on Action Planning and Control

Object weight has been shown to influence visuo-motor planning and control of real grasps (Weir et al., 1991; Brouwer et al., 2006; Eastough and Edwards, 2007). For example, Eastough and Edwards (2007) observed that heavy compared to light objects caused greater peak grip aperture and the opposing placement of the index finger and thumb. This effect of weight on grasping kinematics has been proposed to directly reflect the requirements for a stable grasp (see Smeets and Brenner, 1999 for a review). When grasping heavy objects, to reduce the chances of object rotation and slippage, fingers should be positioned accurately enough so that the grip position passes through the center of mass of the object to be grasped. It is perhaps not surprising, therefore, that weight influences pre-contact kinematics of real grasp movements.

In contrast to real grasps, however, pantomimed grasps entail no preparation for a stable final grip placement on the object. After all, the pretended target is ‘weightless’ and there is no risk of slippage or rotation. The influence of object weight on pantomimed grasps, if any, would thus reflect the pure effort to ‘depict’ the weight of the imagined object by translating a non-spatial property of the object into distinctive features of a motor act.

To determine whether (and to what extent) kinematics of a pantomimed grasp can reveal the weight of the pretended target, in the present study, we first recorded the kinematics of real grasping and pantomimed grasping movements toward differently weighted objects. Using linear discriminant analysis (LDA), we then proceeded to classify the weight of the target – either real or pretended – on the basis of the recorded movement patterns. This innovative approach combining kinematics with classification methods allowed us to obtain a measure of weight-related information transmitted by the hand movements over time.

## Materials and Methods

### Participants

Fifteen participants took part in the study. They had a mean age of 26.8 years (SD: 2.2; range: 24–32 years old; 5 males) and were all right handed, with normal or corrected-to-normal vision, and with no history of either psychiatric or neurological disorders. The experimental procedures were approved by the local ethical committee (ASL 3 Genovese) and were carried out in accordance with the principles of the revised Helsinki Declaration (World Medical Association General Assembly, 2008). Each participant provided written informed consent and was paid in return for participation.

### Apparatus and Procedures

**FIGURE 1:**
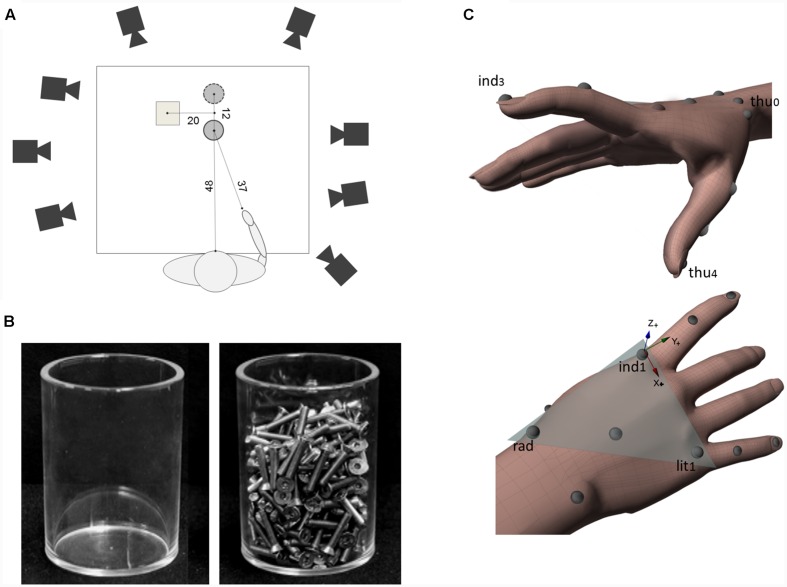
**Experimental set-up and hand models for kinematics parameters computation.** **(A)** A schematic representation of the top view of the experimental set-up (not to scale). The position of the object in real grasp task and in pantomimed grasp task is indicated with a filled and a dashed line circle, respectively. Distances are provided in centimeters. **(B)** A picture of light and heavy object used as target objects. **(C)** The hand model used to compute kinematics parameters together with a graphical representation of the local frame of reference (*Flocal*). *Flocal* had its origin in the marker placed at the metacarpo-phalangeal joint of the index (see *ind1*). Vectors (*ind1 – lit1*) and (*ind1 – rad*) defined the metacarpal plane of the hand (shaded triangle). In this frame of reference, the *x*-axis had the direction of the vector (*ind1 – lit1;* refer to the red arrow) and pointed ulnarly, the *z*-axis was normal to the metacarpal-phalangeal plane, pointing dorsally (refer to the blue arrow), while the *y*-axis was calculated as the cross-product of *z*- and *x*-axes, pointing distally (refer to the green arrow).

Participants were seated on a height-adjustable chair with the right elbow and wrist resting on a table, the forearm pronated, the arm oriented in the parasagittal plane passing through the shoulder, and the right hand in a semi-pronated position, with the tips of the thumb and index finger placed, in gentle opposition, on a tape-marked point. This posture as well as the angular orientation of the wrist were controlled so as to guarantee the consistency of the start position across participants. The working space was set on the surface of a table (width = 140 cm; length = 70 cm; see **Figure 1A**) covered with a black cloth. A glass (height = 11 cm; diameter = 8 cm) was presented on each trial. Depending on the condition, the glass could be empty (i.e., light object; weight = 139 g; see **Figure 1B**) or filled with iron screws (i.e., heavy object; weight = 838 g; see **Figure 1B**).

In the ‘real grasp’ task, participants were requested to reach toward, grasp, pick up either the empty or filled glass, and place it on a platform (height = 7 cm; width = 9 cm; length = 9 cm), located to the left of the target; see **Figure 1A**). The glass was positioned at a distance of about 48 cm from the participant’s body midline with which it was aligned. The angle between the sagittal plane passing through the object and the hand start position was equal to about 35° (see **Figure 1A**).

In the ‘pantomimed grasp’ task, the glass, either empty or filled, was positioned at a displaced location (see **Figure 1A**; dashed line circle). Participants were instructed to imagine that an identical glass was positioned at the target position and were asked to pretend to perform the very same action sequence toward the imagined glass (for a similar paradigm see Goodale et al., 1994).

In both real and pantomimed grasp tasks, participants started the reach-to-grasp movement after a verbal signal from the experimenter. They were instructed to return to the start position and resume hand posture once they were finished placing the glass (or the pretended glass) over the platform. Then, the experimenter returned the glass (if any) to the target position. To ensure that the position of the target object did not vary from trial to trial, for both tasks the glass was placed in between two short pegs that were fixed at the table, the distance between the center of the glass in the real and the pantomimed grasp task being equal to 12 cm (see **Figure 1A**).

In each experimental session, a total of 96 trials were administered in eight separate blocks of 12 trials, i.e., two for each type of movement by object weight combination. Blocks were presented in a fixed order. For each object weight, participants performed the real grasp task followed by the pantomimed grasp task. This was done to allow actual experience with object weight and to prevent spurious weight crossover effects when transitioning from the real grasp to the pantomimed grasp task. The order of presentation of object weight was counterbalanced across participants. On average, the time between trials was 15 s and that between the blocks was 90 s.

At the beginning of each block, the position of the glass (either target or displaced) signaled participants the type of action to be performed (real vs. pantomimed grasp, respectively). Before the experimental session, participants completed 12 practice trials (in four blocks of three trials for each object weight and type of action combination). Block order within the practice session was the same as that adopted during the experimental session. A 2 min pause was allowed between the practice and experimental session. The entire experiment lasted about 60 min.

### Movement Recordings and Kinematics Parameters

To track the kinematics of the hand, we used a near-infrared camera motion capture system (frame rate: 100 Hz; Vicon System). Eight cameras were placed at a distance of 1.5–2 m from the table on which the object was placed. Each participant was outfitted with 13 light-weight retro-reflective hemispheric markers (4 mm in diameter) to create a hand model for kinematics analysis. Markers were placed on the dorsal aspect of the hand and the radial and the ulnar aspect of the wrist. Additional markers were placed at the tip, the metacarpo-phalangeal joint, the phalangeal-phalangeal joint of thumb, the index finger and the little finger, and on the trapezium bone of the thumb (**Figure 1C**).

After data collection, each trial was individually inspected for correct marker identification and then run through a low-pass Butterworth filter with a 6 Hz cutoff. We used a custom software (Matlab; MathWorks, Natick, MA, USA) to obtain the following kinematics parameters:

*grip aperture*, defined as the distance between the marker placed on thumb tip and that placed on the tip of the index finger (mm; see **Figure 1C**);
*wrist velocity*, defined as the module of the velocity of the wrist marker (mm/sec; see *rad* in **Figure 1C**);
*wrist height*, defined as the z-component of the wrist marker (mm).

All these variables were expressed with respect to the original frame of reference (i.e., the frame of reference of the motion capture system, termed as global frame of reference; *Fglobal*). In addition, the trajectory of the index and thumb finger were computed within a local frame of reference centerd on the hand (i.e., *Flocal*; see Ansuini et al., 2015; but also Carpinella et al., 2006, 2011 for a similar method). *Flocal* had its origin in the marker placed at the metacarpo-phalangeal joint of the index finger (see *ind1* in **Figure 1C**).Vectors (*ind1 – lit1*) and (*ind1 – rad*) defined the metacarpal plane of the hand (refer to the shaded triangle in **Figure 1C**). In this frame of reference, the x-axis had the direction of the vector (*ind1 – lit1*) and pointed ulnarly, the *z*-axis was normal to the metacarpal plane, pointing dorsally, while the *y*-axis was calculated as the cross-product of *z*- and *x*-axes, pointing distally (see **Figure 1C**). Within this *Flocal*, we computed the following parameters:

*x-, y-, and z-thumb*, defined as x-, y-, and z-coordinates for the marker placed on the tip of the thumb (mm);
*x-, y-, and z-index*, defined as x-, y-, and z-coordinates for the marker placed on the tip of the index finger (mm);

All these kinematics variables were expressed with respect to normalized (%) rather than absolute (ms) movement durations. To this aim, we first computed time of *reach onset* (i.e., the first time point at which the wrist velocity crossed a 20 mm/sec threshold and remained above it for longer than 100 ms) and time of *reach offset* (i.e., the time at which the wrist velocity dropped below a 20 mm/s threshold) to calculate movement duration (i.e., the time interval between *reach onset* and *offset*). In line with previous evidence (Goodale et al., 1994), analyses revealed that pantomimed movements were longer than real movements (average ± SE: 944 ± 55 vs. 889 ± 42 ms; *p* < 0.05). Moreover, heavy compared to light target elicited longer movement durations (average ± SE: 946 ± 52 vs. 887 ± 44 ms; *p* < 0.05). Of interest, the effect of weight was identical in both real and pantomimed grasps (average ± SE: 910 ± 51 vs. 978 ± 59 ms and 864 ± 39 vs. 914 ± 47 ms for light vs. heavy object in pantomimed and real movements, respectively; *p* > 0.05 for ‘Weight’ by ‘Condition’ interaction). After normalizing the duration of each grasping movement, the data were resampled at intervals of 0.1 of the normalized movement time (resulting in decile increments of normalized reach duration).

To control for outliers, we *z*-transformed normalized data for each condition. Data points with *z-scores* less than -2.5 or greater than 2.5 were classified as statistical outliers and removed. Missing and outlier values (<1.5%) were then replaced using Matlab File Exchange submission inpaint_nans^1^ This procedure interpolates and extrapolates based on sparse linear algebra and partial differential equations (PDE) discretization. A default method was used to solve approximations to PDEs using least squares approach in case of interpolation, while a linear behavior was applied for extrapolation (for a similar procedure see Ansuini et al., 2015).

### Statistical Analyses

#### Principal Component Analysis (PCA) of Kinematic Parameters

To perform dimensionality reduction while retaining the maximum variation present in the original dataset and handling data collinearity (Næs and Mevik, 2001), we performed a principal component analysis (*PCA*) on the set of 90 variables, comprising the 9 spatial features (i.e., *grip aperture*, *wrist velocity*, *wrist height*, *x-, y-, z-thumb*, and *x-, y-, z-index*) across the 10 equally spaced temporal steps of the normalized reaching duration, for 1380 movements (60 over 1440 trials were discarded due to problems related to data recording). Principal Components (PCs) were extracted from a dataset where participants’ data were pooled together rather than separated, thus applying the rule of thumb of higher observations per observed variable ratios in order to get more stable estimates (see Leonard, 2010). Furthermore, Bartlett’s test of sphericity and Kaiser-Meyer-Olkin (KMO) measure of sampling adequacy were used to test for factorability (Bartlett, 1950; Kaiser, 1974). Both tests indicated that the sample was adequate for *PCA* (Bartlett’s test: χ^2^ = 410925,33; d.f. = 4005; *p* < 0.001 and KMO = 0.828).

Mathematically, PCA consists of an orthogonal transfor mation which converts a set of *p* variables – x_1_, x_2_,....x_p_ of a matrix X (where variables are arranged in columns and observations are present in the rows) into p new uncorrelated PCs, *Z* = z_1_, z_2_,....z_p_. The PCs obtained are mutually uncorrelated in the sample and are arranged in decreasing order of their explained sample variances. The PC model, thus, transforms a data matrix X to a second matrix of PC scores, Z as Z = U^t^X, where the columns of *U* = u_1_, u_2_,....u_p_ are the loading vectors, that is, the eigenvectors of the correlation matrix. In our case, the original data matrix X comprised of 90 kinematics variables in a time normalized domain (sampled at each 10% from 10% up to 100% of the movement duration) constituting the variables (columns) while all the 1380 trials from all the subjects were the observations (rows). To simplify data interpretation, we applied a varimax rotation to Principal Component axes to maximize the sum of the variances of the squared coefficients within each eigenvector (Kaiser, 1958). Kaiser’s eigenvalue larger-than-one rule was applied to determine the number of significant components (Kaiser, 1960). The *PCA* led to the selection of the first 13 PCs as significant based on the selection of eigenvalues above 1. To obtain the lower dimension matrix based on the significant PCs, we generated component scores. Component scores are transformed variable values based on the constituent variables and their relative importance for a particular PC.

Mathematically, let i = 1,…, *N* index the rows (observations) and *j* = 1,…, *M* index the columns (variables), then component score for a principal component *k* for observation row *i*, (*Z*_k,i_) can be represented as:

$$Z_{k,i}=u_{i1}*X_{i1}+u_{i2}*X_{i2}+......u_{iM}*X_{iM}$$

The component scores, thus, are a linear combination of the optimally weighted observed variables (Harman, 1976). This allowed us to obtain a lower dimension data set of component scores for all the PCs, with as many rows as original observations (i.e., 1380) and as many columns as the number of significant *PCs* (i.e., 13 *PCs*).

#### Analysis of PCA Data using Linear Discriminant Analysis

To determine the extent to which *PCA* data supported discrimination between the different movement categories, we submitted the output of the *PCA* to a LDA model (see Calder et al., 2001 for a similar procedure). Discriminant functions maximize the ratio of the between group variance (**B**) to the within group variance (**W**), in our instance, the groups being each of the four types of movements (i.e., real grasp_light object, real grasp_heavy object, pantomimed grasp_light object, pantomimed grasp_heavy object). The discriminant functions *y*_i_ are computed from the eigenvectors *l*_i_ of the ratio **W^-1^B** of the between group covariance matrix (**B**) to the within group covariance matrix (**W**):

$$y_{i}=l_{i}\,\;v$$

where *v* is the thirteen-dimensional vector of component scores. The relative size of each eigenvalue (*l*_i_) indicates the relative importance of each of the discriminant functions; rank-ordered according to the size of *l*_i_. The canonical discriminant function coefficients obtained from the eigenvectors express the contribution of each dependent variable to the different discriminant function (Field, 2013). Canonical *R*^2^ (obtained by squaring canonical correlation for each discriminant function) was used as a measure of effect size (Field, 2013).

In *LDA*, the knowledge of the data class labels is used to find a low-dimensional representation that preserves the class differences, so that a classifier can be designed in the feature domain (Nenadic, 2007). For each of the four groups, we determined the location of the point representing the mean for all variables in the multivariate space defined by the variables in the model (i.e., centroids) and then computed the Mahalanobis distances (of the respective case) from each of the group centroids. Therefore, each case was classified as belonging to the group to which it was closest (i.e., where the Mahalanobis distance was smallest). A leave-one-out cross-validation method was applied to evaluate the performance of the *LDA* model (Efron, 1982). In each round of this procedure, one case is held out from the dataset and assigned as a test for the classifier developed by using the remaining cases assigned as training set. This process is repeated until all the withheld cases in the dataset are validated and allows us to calculate the overall diagnostic accuracy of the *LDA* model. To investigate whether allocation distributions differed between expected (i.e., prior probabilities) and observed distributions (i.e., actual group membership), we applied *Chi-squared* test. Finally, to test whether classification scores significantly exceeded chance level, we randomly permuted the class labels and recomputed classification performance and a 95% confidence interval (see Tritchler, 1984; Good, 2005; as implemented by a R package PredPsych^2^ written in R; R Core Team, 2015). All analyses (except permutation testing) were performed using SPSS statistical software (version 21.0).

## Results

### Extracting Principal Components

**FIGURE 2:**
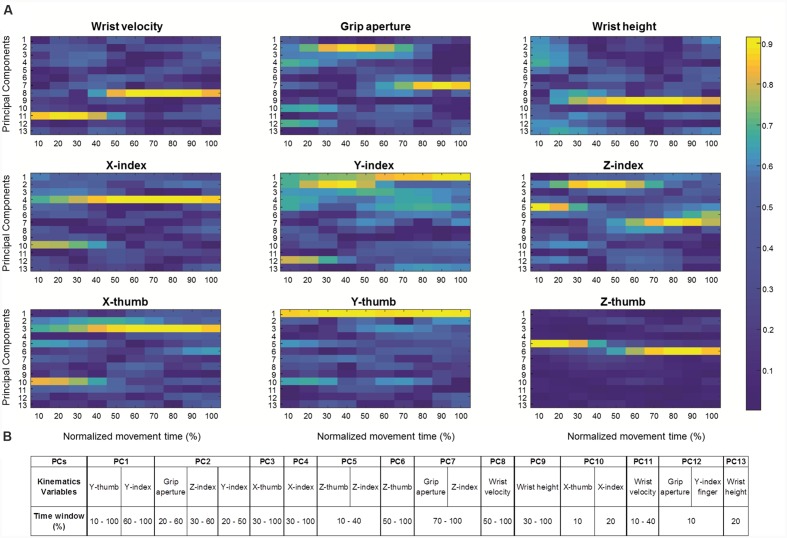
**Factor Loadings for significant Principal Components.** **(A)** Graphical representation of factor loadings across all trials from all participants (heat maps) for the 13 Principal Components (PCs) for each kinematics variable (i.e., wrist velocity, grip aperture, wrist weight, x-, y-, and z-coordinate for both index finger and thumb) over normalized movement time (from 10% up to 100% in 10 step of 10%). Note that factor loadings greater than 0.8 are considered to load significantly on the component. **(B)** A table summarizing the kinematic parameters encoded by each of the 13 Principal Components (PCs), together with the time window in which they are mainly involved.

Thirteen *PCs* having eigenvalues above 1.00 accounted for 92% of the variance and all had communalities (i.e., amount of variance each component has in common with the set of all components; Field, 2013) greater than 0.70 (Dunteman, 1989; Stevens, 1996). It is a general rule to interpret variables with larger factor loadings as representative of the component (Hair et al., 1998). Here we followed this rule and considered factor loadings greater than 0.8 to load significantly on the component. Moreover, if the same variable loaded significantly onto more than one component, we considered the highest factor loading for interpreting the variable contribution on the corresponding component. A graphical representation (heat map) of all factor loadings (i.e., the factor loadings across all trials from all participants) for each variable is reported in **Figure 2**.

As can be seen, for the first seven PCs, high loadings (>0.8) were found only for grip aperture and finger coordinates, suggesting that these PCs were related mainly to the distal aspect of the movement. In particular, the main contribution to *PC1* originated from y-thumb (from 10% to 100% of normalized movement duration) and y-index (from 60% to 100% of movement duration). Grip aperture between 20% and 60% of movement duration, z-index between 30% and 60% of movement duration, and y-index between 20% and 50% of movement duration loaded significantly on *PC2*, while x-thumb from 30% up to 100% of movement duration contributed significantly to *PC3*. For *PC4*, *PC5*, and *PC6*, higher factor loadings were found for x-index between 30% and 100%, z-thumb, and z-index finger at the beginning of the movement (i.e., from 10% up to 40%), and for z-thumb from 50% up to 100% (**Figure 2**). Grip aperture between 70% and 100%, and z-index within the same temporal interval loaded significantly on *PC7*. In contrast, kinematics parameters related to more proximal aspects of the movement were found to load significantly onto *PC8*, *PC9*, and *PC11* (see **Figure 2**). In particular, wrist velocity from 10% up to 40% and from 50% up to the end of the movement contributed to *PC11* and *PC8*, respectively, and wrist height from 30% up to the end of the movement loaded on *PC9*. Finally, an inspection of the factor loadings of *PC10* and *PC12*, revealed large loadings of x-thumb and x-index at 10 and 20% of movement duration on *PC10*, grip aperture and y-index finger at 10% on *PC12*, and wrist height at 20% on *PC13*, suggesting that these components were associated mainly with the earliest phases of the movement.

### Identifying the Discriminant Functions for Different Movement Categories

**FIGURE 3:**
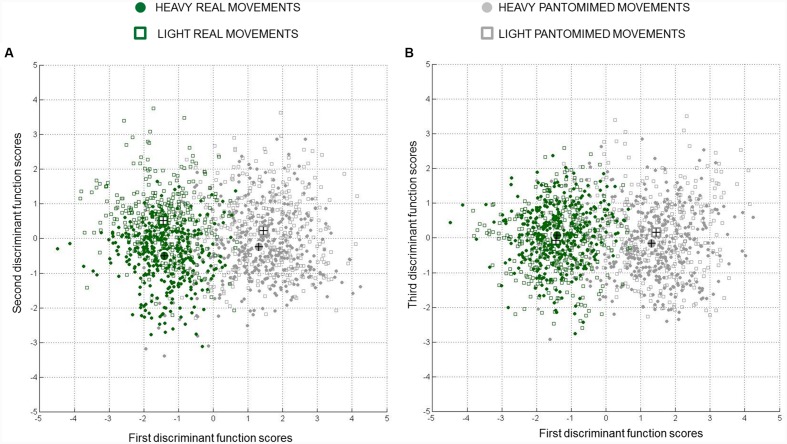
**Combined-group plots for centroids and canonical discriminant function scores.** Group centroids (bigger circles and squares) and individual scores (smaller circles and squares) for **(A)** the first vs. second discriminative functions, and **(B)** the first vs. third discriminative functions are represented. The *x*-axis shows that the first function separated real vs. pantomimed grasps, whereas the *y*-axis shows that the second and the third functions separated the movements toward heavy and light object. Note that cases near a centroid are predicted as belonging to that group. Data for all participants in the sample are presented.

The LDA revealed that the first function accounted for 92.2% of the discriminating ability of the discriminating factors (eigenvalue equal to 2.085; canonical *R*^2^ = 0.68), the second function for 7.1% (eigenvalue equal to 0.162; canonical *R*^2^ = 0.14), and the third function for the remaining 0.7% (eigenvalue equal to 0.016; canonical *R*^2^ = 0.02). As indicated by the *chi-square tests* performed on Wilks’s lambda values (λ value = 0.275; for 1^st^st to 3^rd^ function, 2^nd^nd to 3^rd^ function, and 3^rd^ function, respectively), the combination of the three functions provided a significant discriminative power (*p* < 0.05). A similar result was also found when considering the combination of the second and the third function as well as the contribution of the third function alone (λ value = 0.847; and 0.984; *p*_s_ < 0.05). **Figure 3A** represents the canonical discriminant function scores for each observation, grouped according to the experimental condition to which that observation belonged. This graph, together with the values of the centroids, provides an intuitive visualization of how each function discriminates groups (Field, 2013). As apparent from this figure (please refer to *x*-axis), the first discriminant function mainly separated real and pantomimed grasping movements. The examination of the canonical discriminant function coefficients suggests that this function was most dependent on *PC7*, *PC5*, and *PC9* (please refer to **Table 1**).

**Table 1:** Canonical discriminant function coefficients for the three discriminant functions together with information related to the original kinematics variables that contributed the most to each principal component (PC).

Original features contributing to principal component (PC)	PC number	1^st^ function	2^nd^ function	3^rd^ function
Y-thumb (from 10% up to 100%) and y-index (from 60% up to 100%)	***PC1***	-0,479	-0,240	**0,889**
Grip aperture (from 20% to 60%), z-index (from 30% to 60%), and y-index (from 20% to 50%)	***PC2***	3,878	**0,279**	-0,763
X-thumb (from 30% up to 100%)	***PC3***	-1,067	**0,878**	-0,478
X-index (from 30% to 100%)	***PC4***	-1,726	-0,692	**1,177**
Z-thumb and z-index finger (from 10% up to 40%)	***PC5***	**1,561**	-0,988	-0,785
Z-thumb (from 50% up to 100%)	***PC6***	1,735	**2,094**	1,251
Grip aperture and z-index (from 70% to 100%)	***PC7***	**-3,594**	1,077	-1,011
Wrist velocity (from 50% up to100%)	***PC8***	4,062	1,557	**0,544**
Wrist height (from 30% up to 100%)	***PC9***	**0,325**	0,260	0,301
X-thumb (at 10%) and x-index (at 20%)	***PC10***	3,740	**0,452**	-0,714
Wrist velocity (from 10% up to 40%)	***PC11***	0,220	**0,342**	2,247
Grip aperture and y-index finger (at 10%)	***PC12***	0,736	1,480	**-1,746**
Wrist height (at 20%)	***PC13***	0,140	-0,668	**1,481**


*Values in bold refer to dependent variables for which a significant canonical function correlation coefficient was found. Dependent variables with high canonical function correlations are usually interpreted as contributing the most to group separation (Bargman, 1970).*

**FIGURE 4:**
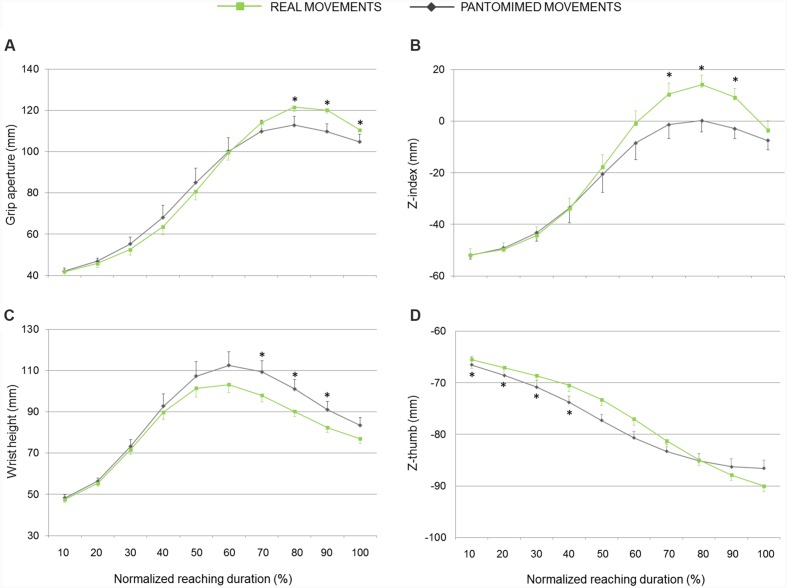
**Hand kinematics of real and pantomimed grasping movements.** **(A)** Grip aperture, **(B)** z-index finger, **(C)** wrist height, and **(D)** z-thumb over time for real (green lines) and pantomimed (gray lines) grasping movements. Data are averaged across trials and participants. Bars represent standard error of the mean. Asterisks refer to statistical significance (*p* < 0.05).

As evident from **Figures 2B** and **3**, the z-coordinate for index and thumb posture, wrist height, and grip aperture contributed the most to *PC7*, *PC5*, and *PC9* so that these kinematics parameters were relatively more important than others for classifying the *reality* of the movement. It is worth noticing, however, that it is difficult to determine which kinematics behavior a PC is coding by simply inspecting the visual representation of its loadings. To complement this visual inspection of kinematics parameters across conditions over time, comparisons of interest were further explored by means of *post hoc* tests (with Bonferroni’s correction). As shown in **Figure 4A**, for what concerns the first discriminant function, grip aperture was greater for real than for pantomimed grasping movement between 80% and 100% of normalized movement duration (*PC7*). Moreover, the index finger was less extended in the palmar direction (i.e., z-index) in real than in the pantomimed movements from 70% up to 90% of normalized movement duration (*PC7*; **Figure 4B**). From 70% up to 90% of the reach-to-grasp movement, the wrist was higher when the movement was pantomimed rather than when it was real (*PC9*; **Figure 4C**). Finally, during the first part of the reaching movement (i.e., from 10% up to 40% of normalized movement duration), the thumb extended more dorsally (i.e., z-thumb) when the movement was real than when it was pantomimed (*PC5*; **Figure 4D**).

**FIGURE 5:**
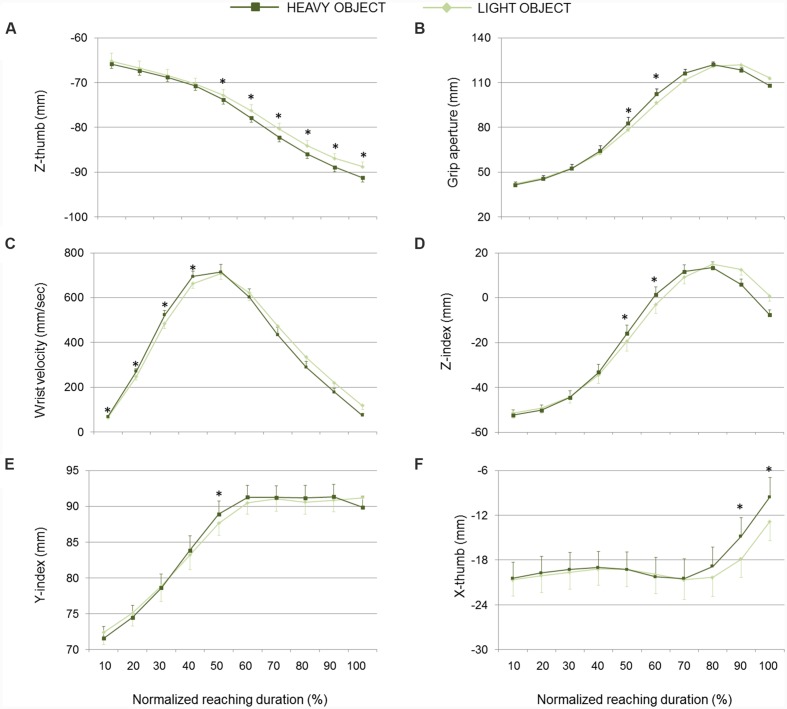
**Hand kinematics of real grasping movements toward light and heavy objects.** **(A)** Z-thumb, **(B)** grip aperture, **(C)** wrist velocity, **(D)** z-index finger, **(E)** y-index finger, and **(F)** x-thumb over time for movements toward heavy and light object (dark and light green lines, respectively). Data are averaged across trials and participants. Bars represent standard error of the mean. Asterisks refer to statistical significance (*p* < 0.05).

**FIGURE 6:**
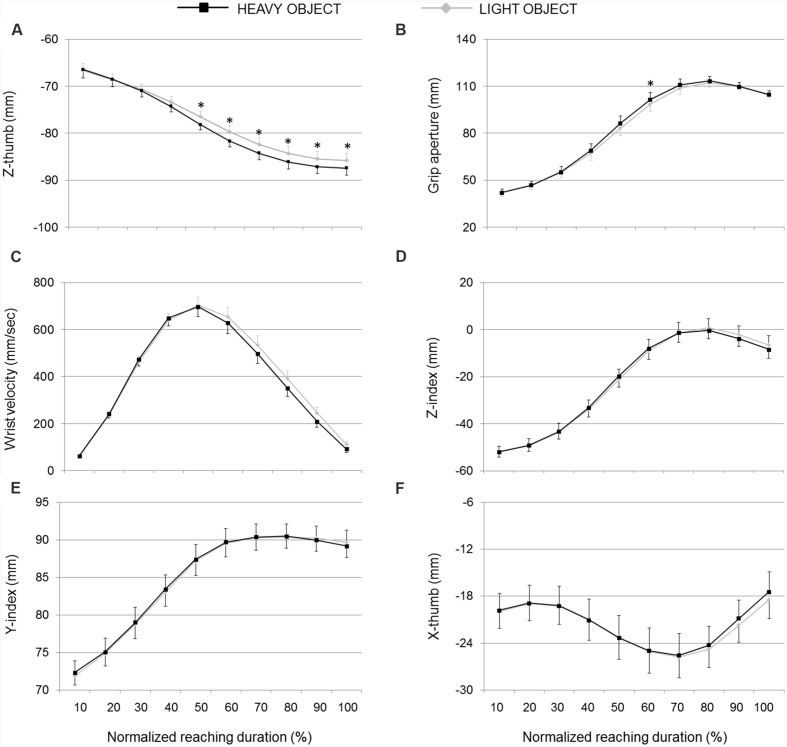
**Hand kinematics of pantomimed grasping movements toward pretended light and heavy objects.** **(A)** Z-thumb, **(B)** grip aperture, **(C)** wrist velocity, **(D)** z-index finger, **(E)** y-index finger, and **(F)** x-thumb over time for movements toward heavy and light object (black and light gray lines, respectively). Data are averaged across trials and participants. Bars represent standard error of the mean. Asterisks refer to statistical significance (*p* < 0.05).

As illustrated in **Figure 3A**, the second discriminant function was more related to weight, supporting separation between real grasp movements performed toward heavy and light objects and, to a less extent, separation between pantomimed movements toward pretended heavy and light targets (please refer to *y*-axis in **Figure 3A**). The *PC6*, *PC3*, *PC10*, *PC11*, and *PC2* correlated significantly with this second function (please refer to **Table 1** for canonical discriminant function coefficients). Examining the kinematics parameters coded by these components (see **Figure 2**) revealed that, for both real and pantomimed movements, the thumb extended more dorsally (z-thumb*; PC6*) during the second phase of the movement when the target was heavy than when it was light (see **Figures 5A** and **6A**, respectively). Moreover, at about half of the reach-to-grasp movement, the grip aperture was smaller when the target was light than when it was heavy (*PC2*; see **Figures 5B** and **6B**). Other variables only expressed weight-related differences for real grasps. For example, wrist velocity between 10 and 40% of normalized movement time was greater for the heavy than for the light object for real but not for pantomimed movements (*PC11*; please refer to **Figures 5C** and **6C**). Similarly, for real grasps but not for pantomimed grasps, the index finger was less extended in palmar direction and pointed more distally (z-index and y-index; *PC2)*, and the thumb pointed more radially (x-thumb; *PC3*), for the heavy object than for the light object **Figures 5D–F** and **6D–F**, respectively). Finally, since the x-coordinate of index finger (*PC10*) did not express significant weight-related differences in either pantomimed or real movements (*p*_s_ > 0.05), no clear interpretation for corresponding component was possible.

For what concerns the third discriminative function, the inspection of **Figure 3B** suggests that this function separated cases based on the weight of the target object. Interestingly, the inspection of centroids suggests that the separation along this function was more pronounced for pantomimed grasps than for real grasps (‘real grasp’ = -0.070 and -0.078 for light object and heavy object, respectively, and ‘pantomimed grasp’ = 0.154 and -0.167 for light object and heavy object, respectively; see **Figure 3B**). This function, however, accounted for only a marginal portion of 0.7% of the total variance. Caution is therefore needed when interpreting the kinematics parameters coded by the correlated *PCs* (*PC12, PC4, PC13, PC1*, and *PC8;* please refer to **Table 1** for canonical discriminant function coefficients).

### Classification of Object Weight

**Table 2A** reports the confusion matrix for the LDA model from the set of *PCA* data. As can be seen, in each of the four categories, reach-to-grasp movements were classified with above chance accuracy (χ^2^_(9)_ = 1.207,8; *p* < 0.05 with an *a priori* probability equal to 25%). In particular, for real grasps, movements toward light and heavy objects were correctly classified on 68 and 67% of cases, respectively, whereas for pantomimed grasps, correct classification of movements toward pretended light and heavy objects occurred in 53 and 49% of cases, respectively.

**Table 2:** Confusion matrix from linear discriminant analyses for **(A)** the four movement type by object weight categories and **(B)** the two object weight categories for real and pantomimed movements separately, applied to the sets of PCA data (i.e., 13 Principal Components).

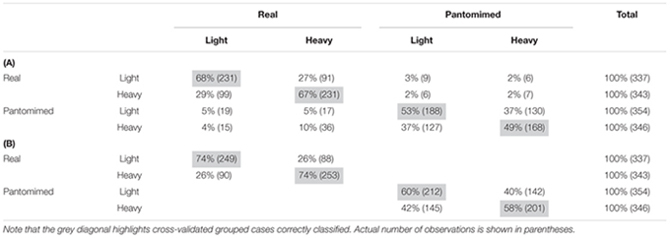

However, since the probability of light vs. heavy classification interacts with the probability of real vs. pantomimed classification, these results might overstate the effect of object weight. In order to adopt a more conservative approach to quantify the impact of weight, we therefore proceeded to perform two separate LDAs for real and pantomimed grasps. **Table 2B** reports the confusion matrices for these analyses. Although the overall proportion of correct classification suffered, classification of object weight was still significantly above chance level for both real and pantomimed grasps. To further support this conclusion, we also performed permutation tests to assess whether correct classification scores were significantly above chance level. By randomly permuting the class labels and recomputing classification performance, we confirmed that the classification scores were indeed significant [(*p*_s_ < 0.001), 95% Confidence Intervals: All four movements (0%, 27%); light/heavy for real movements (0%, 54%); light/heavy for pantomimed movements (0%, 53%) (Tritchler, 1984; Good, 2005)].

## Discussion

Previous research on the relationship between reach-to-grasp movement and the properties of the to-be-grasped object indicates that object weight influences pre-contact kinematics in preparation for a stable final grip placement on the object (Weir et al., 1991; Brouwer et al., 2006; Eastough and Edwards, 2007). Heavy compared to light objects cause increased peak grip aperture, a final finger and thumb placement on the object that more closely passes through the center of mass of the object, and a reduced peak lift velocity (Eastough and Edwards, 2007). Our results confirm and extend these findings by showing that early on in the movement, hand kinematics of real grasps is already scaled to the weight of the to-be-grasped object. As shown in **Figure 5**, the thumb extended more dorsally when the target was heavy than when it was light. Moreover, early on in the reach, grip aperture was larger and wrist velocity was higher for heavy than for light objects. As shown by LDA, prior-to-contact kinematics conveyed indeed enough information to discriminate between real grasp movements aimed at heavy and light objects.

Remarkably, when we examined pantomimed grasp, we found that classification accuracy for heavy vs. light object was lower, but still significantly above the chance level. As for real grasp movements, in the last part of the movement, the thumb extended more dorsally (z-thumb) when the pretended target was heavy than when it was light. Other kinematics parameters sensitive to object weight for real grasp movements, however, showed no similar weight-attunement for pantomimed grasp. For example, whereas the thumb pointed more ulnarly and the index finger pointed more radially for real grasps aimed at a heavy object, no similar modulation was observed for pantomimed movements. In the following, we examine in some details three factors that may have contributed to the differential modulatory effect of weight on real and pantomimed grasps.

A first factor to consider is the removal of the physical object *per se*. During real grasps, the mechanical properties of the object (such as its weight) are critical for motor control. During pantomime, in contrast, the participant’s hand does not come into contact with a material object, but only with ‘thin air.’ Without actual interaction between the hand and the target, there are no obvious consequences for an inaccurate grasping (e.g., the slippage or the roll of the object), permitting one to neglect motor programs that adapt the hand to the material object. This could explain the reduced attunement to weight for pantomimed in comparison to real grasps.

A second factor – causally related to first – refers to the specific role of haptic-based information in sensorimotor transformations supporting prehensile actions. Interestingly, whereas haptic feedback is *per se* not sufficient to evoke motor programs for correct tool use (Goldenberg et al., 2004), there is evidence that removing haptic feedback shifts the response mode from a real one toward a pantomimed one. Even when the movements are directed toward a visible virtual target (viewed in a mirror), removing haptic feedback has been shown to influence grasp kinematics such that grasps without haptic feedback are statistically indistinguishable from pantomimed grasps (Whitwell et al., 2015). The fundamental role of haptic feedback in hand tuning is further supported by evidence from DF, a patient who suffered from visual form agnosia (Schenk, 2012). By using a mirror-apparatus to dissociate the image of an object from its physical presence, it was shown that, without haptic feedback, DF’s grasping performance was not better than her (poor) performance in the manual estimation task (i.e., matching the distance between the thumb and the index finger to the size of the object). Crucially, when intermittent haptic feedback was provided, DF’s performance improved (Schenk, 2012). On this account, the patterning of pantomimed grasp would thus reflect the absence of haptic-based object information.

Removal of the physical object or, more specifically, absence of tactile feedback, however, may be not enough to explain the differential features of pantomimed grasps. Pantomime neglects features of the object that are important for manipulation but have little value for discriminating the object, whereas it specifies features that in actual use are determined by the manipulated object.

A third factor to consider relates thus to the deliberate process of demonstrating the properties of the pretended target (Goldenberg et al., 2003). We speculate that the kinematics of pantomimed actions may convey information about the symbolic motor representation of the pretended weight (Goldenberg et al., 2003; see also, Hermsdörfer et al., 2005, 2006; Laimgruber et al., 2005). However, we wish to emphasize that these considerations are of a very speculative nature because participants in our study were not explicitly instructed to communicate the weight of the object. An interesting prediction for future studies is that explicitly instructing participants to communicate object weight to another person should increase weight discriminability for pantomimed grasp.

Related to this, it will be interesting to investigate to what extent observers watching a pantomimed grasp are able to infer the properties of the pretended object. Some behavioral studies already indicate that the weight of an object (e.g., a box) can be inferred quite accurately when observing another person lifting it (Runeson and Frykholm, 1983; Bingham, 1987; Hamilton et al., 2004). Moreover, there is evidence that muscle-specific M1 excitability modulates to the force requirements of observed object lifting (i.e., M1 excitability is considerably higher when observing heavy object lifting compared to light object lifting) and that this modulation is sensitive to the kinematics conveyed by the observed action (Alaerts et al., 2010a,b). To our knowledge, however, no previous study has investigated whether observers are able to read out the weight of a to-be-grasped object from pre-contact kinematics. Moreover, there is no information in the literature regarding observers’ ability to infer object weight from pantomimed grasps. The classification results in our study lend some plausibility to this hypothesis by showing that pre-contact kinematics provide a firm informational basis for weight discrimination for real grasps and – albeit to a lesser extent – for pantomimed grasp. In future research, we plan to test whether and to what extent observers are able to make use of this information to discriminate weight and other non-spatial object properties (such as object fragility). Future research should also focus on the extent to which real and pantomimed grasps convey a categorical representation of weight information (i.e., an object is either heavy or light) versus a continuous representation of weight (i.e., changes in activity patterns that directly correspond to changes in object weight) and on the exact time course of weight specification (i.e., how weight information is specified at specific time intervals).

Finally, it will be important to consider these results from the perspective of the neural mechanism involved in extracting object weight when pantomiming a reach-to-grasp movement. Consistent with the proposed division of labor in the visual pathways of the primate cerebral cortex, between a dorsal pathway specialized for action control and a ventral stream dedicated to the perception of the visual world (Milner and Goodale, 1995), processing object features critical for motor control, such as object weight, has been traditionally thought to be in the purview of the dorsal pathway. Recent functional neuroimaging (fMRI) evidence, however, suggests that, in addition to traditional motor-related areas, the lateral occipital cortex (LOC) in the ventral visual stream represents object weight when preparing to lift an object (Gallivan et al., 2014). Expanding upon this result, it is tempting to speculate that the LOC representation of object weight may inform and support weight-related pantomime. Functional neuroimaging studies and patient studies may help to clarify the differential contribution of the ventral and the dorsal pathways to object weight processing in preparation of real and pantomimed grasps.

## Author Contributions

CA: designed the experiment, analyzed the data and wrote the article. AC: designed and performed the experiment. CC: analyzed the data. AK: performed the experiment and analyzed the data. DQ: performed the experiment. CB: designed the experiment, supervised the work and wrote the article.

## Conflict of Interest Statement

The authors declare that the research was conducted in the absence of any commercial or financial relationships that could be construed as a potential conflict of interest.

## Footnotes

1 http://www.mathworks.com/matlabcentral/fileexchange/4551-inpaint-nans.

2 https://github.com/ateshkoul/PredPsych
